# Diversity of Cellulase-Producing Filamentous Fungi From Tibet and Transcriptomic Analysis of a Superior Cellulase Producer *Trichoderma harzianum* LZ117

**DOI:** 10.3389/fmicb.2020.01617

**Published:** 2020-07-14

**Authors:** Jia-Xiang Li, Fei Zhang, Dan-Dan Jiang, Jun Li, Feng-Lou Wang, Zhang Zhang, Wei Wang, Xin-Qing Zhao

**Affiliations:** ^1^State Key Laboratory of Microbial Metabolism, Joint International Research Laboratory of Metabolic and Developmental Sciences, School of Life Sciences and Biotechnology, Shanghai Jiao Tong University, Shanghai, China; ^2^R&D Center, JALA Group Co., Shanghai, China; ^3^State Key Laboratory of Bioreactor Engineering, East China University of Science and Technology, Shanghai, China

**Keywords:** filamentous fungi, cellulase production, *Trichoderma harzianum*, comparative transcriptome analysis, cellulase induction

## Abstract

Filamentous fungi are widely used for producing cellulolytic enzymes to degrade lignocellulosic biomass. Microbial resources from Tibet have received great attention due to the unique geographic and climatic conditions in the Qinghai-Tibet Plateau. However, studies on cellulase producing fungal strains originated from Tibet remain very limited, and so far no studies have been focused on regulation of cellulase production of the specific strains thereof. Here, filamentous fungal strains were isolated from soil, plant, and other environments in Tibet, and cellulase-producing strains were further investigated. A total of 88 filamentous fungal strains were identified, and screening of cellulase-producing fungi revealed that 16 strains affiliated with the genera *Penicillium*, *Trichoderma*, *Aspergillus*, and *Talaromyces* exhibited varying cellulolytic activities. Among these strains, *T. harzianum* isolate LZ117 is the most potent producer. Comparative transcriptome analysis using *T. harzianum* LZ117 and the control strain *T. harzianum* K223452 cultured on cellulose indicated an intensive modulation of gene transcription related to protein synthesis and quality control. Furthermore, transcription of *xyr1* which encodes the global transcriptional activator for cellulase expression was significantly up-regulated. Transcription of *cre1* and other predicted repressors controlling cellulase gene expression was decreased in *T. harzianum* LZ117, which may contribute to enhancing formation of primary cellulases. To our knowledge, this is the first report that the transcription landscape at the early enzyme production stage of *T. harzianum* was comprehensively described, and detailed analysis on modulation of transporters, regulatory proteins as well as protein synthesis and processing was presented. Our study contributes to increasing the catalog of publicly available transcriptome data from *T. harzianum*, and provides useful clues for unraveling the biotechnological potential of this species for lignocellulosic biorefinery.

## Introduction

Lignocellulose biomass is abundant in nature, and represents more than half of the organic matter produced globally via plant photosynthesis. Bioconversion of relatively inexpensive lignocellulosic biomass into biofuels and value-added products can effectively alleviate pressure of energy supply and benefit sustainable development. At present, industrial cellulase preparations for biomass degradation are generally produced from filamentous fungi (Payne et al., 2015) and comprise a mixture of glycoside hydrolases (GHs) as well as other accessory proteins that are required to work synergistically with cellulases (Champreda et al., 2019).

Tibet is regarded as one of the richest areas for biodiversity in China as a consequence of its complex topography and diverse habitats. The Tibetan plateau ecosystem may be subjected to various environmental stresses characterized by low temperature, anoxia and high solar radiation, which endows unique properties of the microbial strains. However, limited studies have been conducted to identify and evaluate potential lignocellulolytic enzyme-producing fungi from this environment. Previously, diversity of cultivable *Trichoderma* strains in Tibet soil samples and diverse fungal strains from permafrost at Qinghai-Tibet Plateau were reported (Sun et al., 2012; Hu et al., 2014), but cellulase producers were not described. It will be of great interest to explore novel filamentous fungal strains from Tibet for cellulase production and biomass degradation.

*Trichoderma harzianum* is closely related to *T. reesei*, which is widely used for production of cellulolytic enzymes (Bischof et al., 2016; Zhang et al., 2019). Studies on various *T. harzianum* strains showed that they can produce cellulolytic enzyme complex with higher β-glucosidase and endoglucanases activities than that shown by *T. reesei* (de Castro et al., 2010; Benoliel et al., 2013). The secretome of *T. harzianum* and genes encoding carbohydrate-active enzymes in *T. harzianum* were also reported (Rocha et al., 2016; Ferreira et al., 2017; Horta et al., 2018). However, the regulatory mechanisms underlying cellulase production in *T. harzianum* remain unclear. Two related studies have been performed using comparative transcriptome and secretome analysis (Horta et al., 2014, 2018). However, both studies used samples collected at late fermentation stage (96 h). Therefore, information on the regulatory events at early enzyme production stage, especially for cellulase induction, remains to be elucidated. In our previous studies, *T. harzianum* LZ117 was isolated from the surface of a moss collected in Tibet. Compared with the wild-type and mutant strains of the commonly used cellulase producer *T. reesei*, early cellulase induction and high production level of this strain were revealed, suggesting great potential of the strain as a robust cellulase producer (Li et al., 2019). On the other hand, *T. harzianum* LZ117 also serves as a good model to reveal the underlying regulatory mechanisms for cellulase induction and production.

In this report, we present results on isolating filamentous fungal strains from Tibet, with the aim to explore novel cellulase-producing strains. In addition, comparative transcriptome analysis was performed to reveal the mechanisms underlying regulation of cellulase production in *T. harzianum* LZ117. The results here would be helpful to develop better cellulase producers for lignocellulosic biorefinery.

## Materials and Methods

### Description of Sample Collection for Strain Isolation

A total of 36 samples (22 plant samples, 10 soil samples, one melt iceberg water sample from the mountain, two samples from the surface of unknown mushroom and one barley wine starter sample) were collected from different locations in Tibet Autonomous Region of China in August 2018 (details in Supplementary Table S1). The soil samples were taken at a depth of 9–10 cm beneath the earth’s surface and a distance of 2–3 cm from plant rhizosphere. The samples were placed in sterilized polyethylene bags and stored at 4°C for subsequent fungal isolation.

### Isolation of Filamentous Fungal Strains

Serial dilution method was adopted for fungal isolation by using a series of selective media (summarized in Supplementary Table S2). Specifically, each sample (0.5–1 g) was immersed in 5 mL of sterile double-distilled H_2_O, placed in a centrifuge tube with aseptic glass beads and grinded by Scientz-48 High-Throughput Tissue Grinder (Scientz Bio-tech, Ningbo, China), which was followed by serial dilution using sterilized distilled H_2_O. An aliquot of 100 μL of each diluted sample was inoculated onto the Petri-plates containing the selective media supplemented with streptomycin (1%, w/v) to inhibit bacterial growth for primary isolation of fungi. Fungal cultures were incubated at 28°C for 3–5 days, and putative filamentous fungal colonies from the primary isolation agar plates were purified by two rounds of sub-culture on potato-dextrose agar (PDA) and cultured at 28°C. Pure cultures of the fungi were preserved in 20% (w/v) glycerol at −80°C.

### Morphological and Molecular Analysis of the Fungal Strains

Filamentous fungal isolates were identified at the genus level via a combination of morphological properties and molecular analysis. Colony characteristics and microscopic morphology of fungal mycelia cultured on malt extract agar (MEA) or Potato Dextrose Agar (PDA) plates were observed following 7-day growth at 28°C. Molecular identification of filamentous fungal species was based on the analysis of the sequence of ITS (Internal Transcribed Spacer, including ITS1-5.8S-ITS2 region sequence of rRNA gene) previously recommended as a universal barcode to identify fungal species (Schoch et al., 2012).

Extraction of total DNA from fungal mycelia was performed according to the method described previously (Zhao et al., 2016). Briefly, mycelia were grinded with 0.4–0.6 mm glass-beads (Magen Biotech Co., Ltd., Guangzhou, China) in a specific amount of lysate reagent (40 mM Tris-HCl, 10 mM ethylenediaminetetraacetic acid, 20 mM sodium acetate, and 1% sodium dodecyl sulfate, pH 8.0), then centrifuged at 12,000 × *g* for 3 min, followed by transfer of the supernatant into a new centrifuge tube. Subsequently, DNA precipitation was carried out at −20°C by adding isopropanol at a ratio of 1 supernatant: 1 isopropanol (v/v). Genomic DNA was collected after centrifugation at 12000 × *g*, 4°C for 10 min, then washed twice with 70% (v/v) ethanol, and dissolved in 50 μL sterile distilled H_2_O.

ITS region was amplified from genomic DNA by PCR using primers ITS4 and ITS5 (White et al., 1990). The purified PCR products were sequenced by Tsingke Biotech Co., Ltd. (Shanghai, China). Identification of fungal isolates was performed via querying the obtained sequences against the NCBI database using BLASTn^1^. The sequences obtained were deposited in National Centre for Biotechnology Information (NCBI) under GenBank accession numbers from MK804322 to MK804409 (Supplementary Table S1).

### Phylogenetic Analysis of the Fungal Strains

For phylogenetic analysis in MEGA v6.0 software (Tamura et al., 2013), the ITS region sequences of 88 isolates included in 43 filamentous fungal species were used to construct phylogenetic tree according to the neighbor-joining method (NJ tree, Saitou and Nei, 1987) after multiple alignment of sequences data by CLUSTAL_X (Thompson et al., 1997). The corrected evolutionary distance was evaluated according to Kimura’s two-parameter model (Kimura, 1980). In order to estimate the consensus of the branching, the bootstrap resampling analysis of phylogenetic tree was employed with 1000 replicates of the data set.

### Screening of Cellulolytic Fungi by Agar Culture and Submerged Culture

Extracellular cellulolytic activities were measured both qualitatively and quantitatively. For qualitatively evaluation, the fungi were cultivated on MA (Mandels and Andreotti, 1978) solid medium supplemented with bead-milling cellulose (20 g/L, w/v) as a sole carbon source at 28°C for 7 days. Cellulase activities were observed as transparent zones around the fungal colonies.

In regard to quantitative analysis of cellulase activities, 250 mL Erlenmeyer flasks with 50 mL MA medium [with 2% (m/v) lactose added as the carbon source] supplemented with 0.1% (w/v) peptone were inoculated with fungal spores (1 × 10^5^/mL) collected from the culture in MEA medium, and incubated for 48 h at 28°C and shaking at 180 rpm for mycelial germination. Subsequently, the vigorous hyphal cultures were inoculated respectively with an inoculation size of 10% (v/v) into 250 mL Erlenmeyer flasks containing 50 mL MA mediums supplemented with 0.1% (w/v) peptone and 2% (m/v) microcrystalline cellulose (Merck, Germany) as the sole inducer for cellulase production at 28°C, shaking at 180 rpm.

### *T. harzianum* Strains and Growth Conditions

*Trichoderma harzianum* LZ117 was deposited at the China General Microbiological Culture Collection Center (CGMCC) by the strain number CGMCC 17184. *T. harzianum* K223452 (CGMCC 17199) that showed less potent cellulase production profile (Li et al., 2019) was used as the reference strain for comparative transcriptome analysis. The 7-day cultures of the fungal strains grown on MEA plates at 28°C were used to collect conidia. Mature conidia of strains were harvested using 2 mL of sterilized distilled H_2_O and the prepared conidia suspensions were stored in sterilized 20% glycerol at −80°C.

### Enzyme and Protein Concentration Assays

Sampling was performed at an interval of 24 h and the culture supernatants collected via centrifugation (10,000 rpm for 5 min at 4°C) were used for enzyme and secreted protein concentration assays according to the previously described method (Meng et al., 2020). Briefly, the activities of filter paper (FPA), CMCase and xylanases were measured using Whatman No. 1 filter paper (50 mg, 1.0 × 6.0 cm^2^), CMC-Na (Sigma-Aldrich, United States) and beechwood xylan (Sigma-Aldrich) as substrates, respectively. The reaction mixtures contained 50 mg of filter paper, 1.0% CMC-Na or 1.0% xylan with 500 μL of the suitably diluted enzyme fractions. These mixtures were then incubated at 50°C for 60 min (FPA) or 30 min (CMCase and xylanases activities). The amount of reducing sugar released was determined using the DNS method (Ghose, 1987). One unit of enzymatic activity (U) was defined as the amount of enzyme required to produce 1 μmol of reducing sugar per min from the reaction substrates. The activities of cellobiohydrolases (CBHs) and β-glucosidase (BGL) were determined using *p*-nitrophenyl-β-D-cellobioside (*p*NPC) and *p*-nitrophenyl-β-D-glucopyranoside (*p*NPG) (Sigma-Aldrich) as substrates, respectively. The diluted supernatants (100 μL) were incubated with 50 μL of 10 mM *p*NPC or *p*NPG dissolved in 50 mM acetate buffer (pH 5.0) at 50°C for 30 min. Then, 150 μL of each sample was mixed with an equal volume of 10% sodium carbonate to stop the reactions. The absorbance at 405 nm was then measured. One unit of CBHs or BGL activity was defined as the amount of enzyme releasing 1 μmol of *p*NP per minute from the appropriate substrate. Concentration of secreted protein was determined using the Modified BCA Protein Assay Kit (Beyotime Bio-tech, Shanghai, China). All experiments were performed in three biological replicates.

### Quantitative Reverse Transcription-PCR (RT-qPCR) Analysis

Due to unknown genomic context or coding sequences of genes to be detected in the naturally isolated *T. harzianum* strains, the primers for RT-qPCR analysis cannot be designed based on the known sequences. Thus, partial CDS sequences of the selected genes, including several cellulase genes, regulators as well as the internal control gene β-*tubulin*, were acquired by PCR with the primers which were designed according to corresponding CDS sequences from three reference genomes of *T. harzianum* strains (*T. harzianum* TR274 v1.0, *T. harzianum* T22 v1.0, *T. harzianum* M10 v1.0) on the Joint Genome Institute (JGI)-MycoCosm database^2^. Next, the generated CDS fragments were sequenced and aligned by Clustal W^3^, then a length of identical CDS sequence for each selected gene between *T. harzianum* LZ117 and the reference strain *T. harzianum* K223452 was used for designing respective quantitative primers. The primers for cloning of CDS fragments and RT-qPCR analysis are listed in Supplementary Table S3.

For RNA sampling, mycelia were harvested from *Trichoderma* strains that were cultivated on 2% microcrystalline cellulose at 24 h after a shift from lactose. RNA extraction, reverse transcription, and RT-qPCR were performed as reported previously (Meng et al., 2020). The relative transcription of genes was calculated by the 2^–ΔΔCt^ method (Livak and Schmittgen, 2001), with the β-tubulin gene as the internal reference gene for normalization (da Silva et al., 2017). Two biological replicates and three technical replicates for each reaction were carried out.

### Comparative Transcriptomic Analysis

For comparative transcriptomic analysis between *T. harzianum* LZ117 and *T. harzianum* K223452, mycelia of the two strains cultured in MA medium supplemented with 2% microcrystalline cellulose and 0.1% peptone were collected at 24 h, and total RNA was extracted using the Spin Column Plant Total RNA Purification Kit (Sangon Biotech, China). RNA-seq based on Illumina Novaseq 6000 sequencing platform as well as transcriptome analysis was completed by Majorbio Bio-Tech (Shanghai, China) using the standard analysis methods. The reference genome of *T. harzianum* CBS 226.95 (assembly Triha v1.0; taxid: 983964) from NCBI genome database^4^ was applied for mapping the sequenced clean reads using the software HISAT2 (Kim et al., 2015), and six major databases were adopted for gene homology and functional annotations (see Supplementary Table S4). TPM (Transcripts Per Million reads) (Conesa et al., 2016) was used as an index to analyze the gene expression levels by RSEM v1.2.12 software, and differential expressed genes were selected with the DESeq2 software (Love et al., 2014), with the thresholds: *p*-adjust < 0.05 and |Log_2_FC| ≥ 1. CAZymes classification was performed according to the annotation of CAzymes genes in *T. harzianum* CBS 226.95^5^. In addition, the added descriptions of glycoside hydrolase genes and its transcription factor genes were based on the homologues in a model strain *T. reesei* QM6a^6^. Comparative transcriptome data analysis was completed partially using the free online Majorbio Cloud Platform^7^. Two biological replicates were performed, and the transcriptomic data are available at the SRA web site^8^ under the accession number PRJNA613881.

### Statistical Analysis

For each experiment, at least two biological replicates were performed with technical duplicates, and reproducible results were presented. All enzyme or protein assay data shown in this paper were carried out at least three times with identical or similar results. The error bars indicate the standard deviation (SD) from the mean of triplicates. Student’s *t*-test was used to compare two samples, and *p* < 0.05 was considered to be significant.

## Results and Discussion

### Biodiversity and Phylogenetic Analysis of the Isolated Filamentous Fungi

A total of 88 isolates of filamentous fungi (Supplementary Table S1) were isolated from the surface and/or interior of 36 distinct and unique samples collected from Tibet region in China, and the obtained strains were further characterized. Phylogenetic analysis showed that 88 isolates belong to three phyla, six classes, 12 orders, 18 families and 22 genera (Supplementary Figure S1).

**FIGURE 1:**
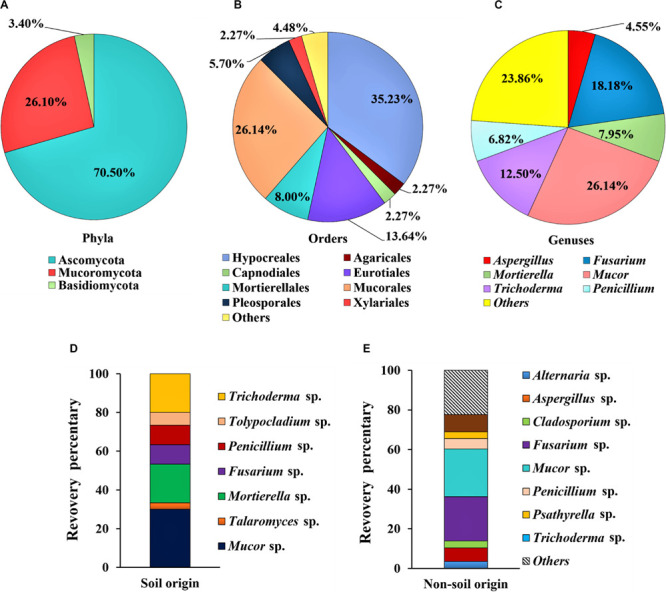
Composition of the dominant filamentous fungal strains isolated from Tibet. The classification was shown at the phyla level **(A)**; order level **(B)**; and genus level **(C)**; and comparison on the diversity of the fungal strains between the soil **(D)** and the non-soil **(E)** samples.

Among the isolates, 70.5% of strains (62 isolates of 19 genera) were identified as *Ascomycota*, while 3.4% strains (three isolates of two genera) were identified as *Basidiomycota* and 26.1% strains (23 isolates of one genus) were identified as *Mucoromycota* (Figure 1A). At the order level, 35.2% of isolates (*n* = 31) belong to *Hypocreales*, 26.1% to *Mucorales* (*n* = 23), 13.6% to *Eurotiales* (*n* = 12), 8.0% to *Mortierellales* (*n* = 7), and 5.7% to *Pleosporales* (*n* = 5) (Figure 1B). At the genus level, over a half of the whole isolated strains were represented by the *Mucor* sp. (26.1%), *Fusarium* sp. (18.2%), and *Trichoderma* sp. (12.5%) (Figure 1C). From the soil samples, a total of 30 fungal strains were obtained, and the dominant groups of genus are *Mucor* sp. (30.0%), *Mortierella* sp. (20.0%) and *Trichoderma* sp. (20.0%); and the other groups are less than 10% for each genus (Figure 1D). In contrast, 58 fungal strains were obtained from samples of non-soil origin, and the most frequently isolated groups are *Mucor* sp. (24.1%), *Fusarium* sp. (22.4%), whereas the other groups are less than 10% for each genus (Figure 1E).

Strains belonging to the genus of *Mucor* are the most abundant fungal strains that were isolated from Tibet in this study. β-glucosidase from *Mucor circinelloides* have been studied (Kato et al., 2013; Huang et al., 2014), and it will be interesting to further study cellulase production from the isolated *Mucor* strains in the future work. Cellulase-producing *Fusarium* strains, including *F. oryzae* and *F. oxysporum* have been reported in the previous studies (Qin et al., 2010; Zhao et al., 2013; Xu et al., 2015). We did not isolate *Mucor* and *Fusarium* strains of the same species, and due to fewer studies on cellulase production from *Mucor* and *Fusarium* strains than that from *Aspergillus*, *Penicillium, Talaromyces*, and *Trichoderma*, we only tested cellulase activities in some selected strains, which are further discussed as below. However, we cannot exclude the possibility that the *Mucor* and *Fusarium* strains presented here have cellulase-producing ability. On the other hand, we identified these fungal strains based on the ITS sequences, but for more accurate species characterization, more house-keeping genes should be sequenced, which will be performed in future studies.

### Cellulase Activity of the Isolated Fungal Strains

Filamentous fungi, particularly, *Penicillium* sp., *Trichoderma* sp., *Aspergillus* sp., are of great interest due to their great capacity to produce plant cell wall-degrading enzymes (PCWDEs) used to convert lignocellulosic biomass to fermentable sugars (Passos et al., 2018). In addition, the *Talaromyces* genus, early regarded as a teleomorph state of *Penicillium*, forms a monophyletic clade distinct from the other *Penicillium* subgenera (Yilmaz et al., 2014), and *Talaromyces* strains have also been studied as cellulase producers (Liao et al., 2018; Méndez-Líter et al., 2019). Thus, 23 fungal strains that belongs to the genera of *Penicillium*, *Trichoderma*, *Aspergillus* and *Talaromyces* were selected for evaluation of PCWDEs production. An initial transparent zone assay based on cellulose solid plates with above-mentioned strains indicated that they all possessed cellulolytic activities (data not shown). Then, a quantitative assay of extracellular enzyme from the cellulolytic fungi was performed. In liquid culture, the selected fungal strains showed varying activities of cellulases, CMCase, *p*NPCase, *p*NPGase, and xylanase (Table 1) in the crude enzyme extracts. It can be seen directly that isolates derived from the same genus showed significant variations in the activities of the exo-cellular enzymes. Three strains, *T. harzianum* LZ117, *Trichoderma* sp. SC56-113 and *Trichoderma* sp. SC13-114, were revealed to produce higher levels of cellulase activities. CBHs (*p*NPCase) production was significantly higher in *Trichoderma* sp. SC56-113, and other strains including *Trichoderma* sp. SC13-114, *Aspergillus* sp. SC3-DY9, and *Aspergillus* sp. SC3-48 also exhibited comparable *p*NPCase activity. Greater CMCase activity was observed in the case of three strains belonging to *T. harzianum* LZ117, SC56-113 and SC13-114 as compared to that from the other strains. Also, *T. harzianum* LZ117 produced significantly higher β-glucosidase activity (*p*NPGase, 2.91 U/mL) compared to the other strains. *Aspergillus* sp. and *Penicillium* (including *Talaromyces*) sp. isolated in this study showed lower level of β-glucosidase activity than that from the *Trichoderma* sp. strains. Furthermore, among these strains, significantly higher xylanase activity (51.87 ± 2.267 U/mL) was observed for *T. harzianum* LZ117. A notable xylanolytic activity was also observed in the case of *Trichoderma* sp. strains SC56-113 and SC13-114. The reason why certain enzyme activities could scarcely be detected in some strains might be due to the limited enzyme secretion capacity under the conditions employed in this study. Another reason is that enzyme measurement conditions used in this study are not optimal. Additionally, the diversity of cellulase and xylanase production detected might be resulted by different mycelial biomass. It is worth noting that *T. harzianum* LZ117 exhibited the most brilliant enzymatic activities, which renders it a potent source of PCWDEs for lignocellulose conversion.

**TABLE 1:** Enzymatic activities of the isolated cellulolytic fungal strains from Tibet*.

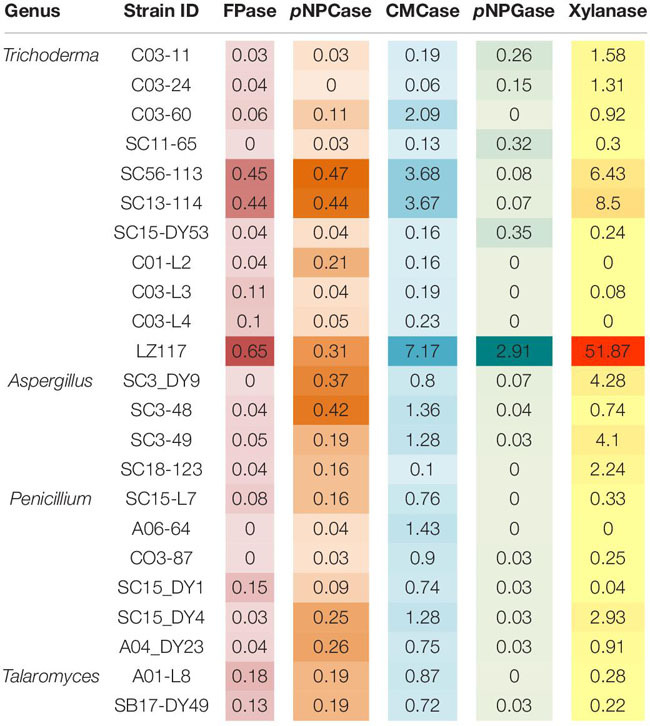

*^∗^The change of table background color from light to dark in each row represents the transition from low enzyme activities to high enzyme activities.*

We assumed that the unique environment such as high altitude in Tibet are inclined to endow local microorganisms special characteristics, including the ability to adapt to low temperature (Hu et al., 2015). It is of great interest to explore cold-adapted cellulase among the fungal strains. Unfortunately, we did not find cold-adapted cellulases using the conditions in this study (data not shown).

### Comparative Transcriptome Analysis

**FIGURE 2:**
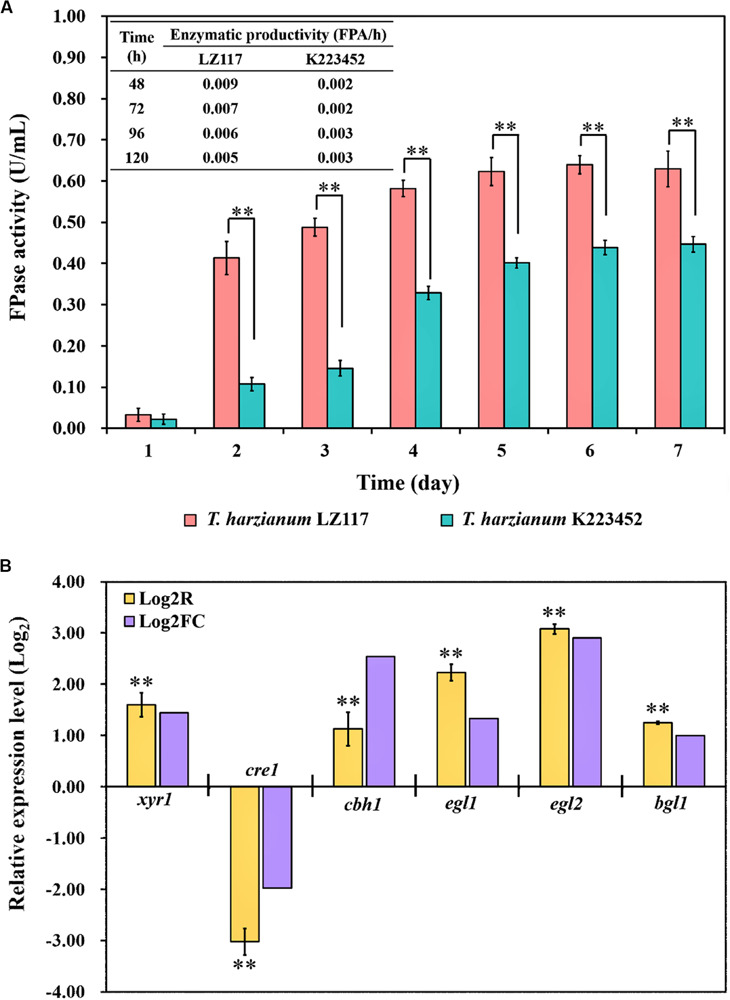
Cellulase production of *T. harzianum* strains and validation of the transcriptome analysis. **(A)** Cellulase production profiles [FPA (U/mL) and productivity (FPA/h)] of *T. harzianum* LZ117 and *T. harzianum* K223452 cultured in shake flask using MA medium supplemented with 0.1% peptone and 2% microcrystalline cellulose. **(B)** Verification of the RNA-Seq data by RT-qPCR analysis using the selected genes encoding cellulolytic enzymes and transcription factors in *T. harzianum* LZ117 at 24 h (Log_2_R and Log_2_FC indicate results for RT-qPCR and RNA-seq, respectively). Error bars show the standard deviations, and the differences between groups were considered significant at *p* < 0.01 (Student’s *t*-test) and are indicated by ^∗∗^.

*T. harzianum* LZ117 presented fast kinetic production of cellulase, which was represented by significantly higher Filter paper activity (FPA) and shorter fermentation time as compared to *T. harzianum* K223452, and FPA enzymatic profiles [productivity (FPA/h)] were shown in Figure 2A. Regulation of cellulase production *T. harzianum* has been limitedly studied so far, to further understand the mechanisms underlying faster cellulase formation and superior enzymatic activities of *T. harzianum* LZ117, comparative transcriptomic analysis for *T. harzianum* LZ117 and *T. harzianum* K223452 under submerged culture for cellulase production was performed. We believe that the mechanism studies would also provide clues to further engineering other cellulase-producing strains isolated from this work.

Due to the consideration that important molecular events mostly happen at early growth stage, we performed the transcriptome analysis using samples collected at 24 h. Transcriptome data showed a high correlation and consistency between the two biological replicates (Supplementary Figure S2). To further verify the reliability of transcriptome data, gene transcription analysis of *T. harzianum* LZ117 over *T. harzianum* K223452 at 24 h was performed by RT-qPCR for several genes, including the transcription factor genes *xyr1* and *cre1*, as well as glycosyl hydrolase genes *cbh1* (*cel7a*), *egl1* (*cel7b*), *egl2* (*cel5a*), and *bgl1* (*cel3a*). The RT-qPCR analysis results reflected that the changing trend in transcriptional levels of these genes was in consistency with those obtained from the comparative transcriptome analysis (Figure 2B), indicating that the RNA-seq data are reliable.

#### Functional Enrichment Analysis of Differentially Expressed Genes (DEGs)

A total of 10887 unique transcripts were detected by RNA-seq analysis. Comparative transcriptome analysis showed that there were 1160 DEGs between *T. harzianum* LZ117 and K223452 at 24 h of cellulose induction (|log2FC| ≥ 1 and adjusted *p*-values ≤ 0.05), of which 375 genes were up-regulated and 785 genes were down-regulated (Supplementary File S1).

**FIGURE 3:**
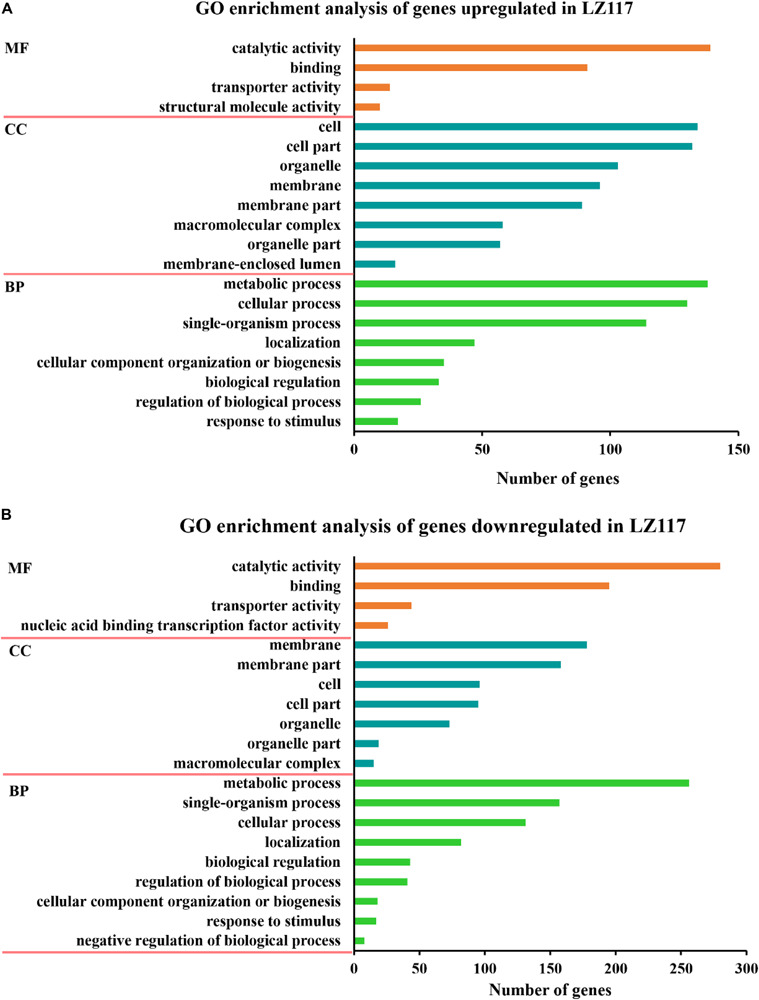
GO enrichment analysis of the changed genes. Results of GO enrichment analysis of the upregulated **(A)** and downregulated **(B)** genes in *T. harzianum* LZ117 relative to *T. harzianum* K223452 were presented. The Y axis represents the name of the most enriched GOs: (MF) molecular function, (BP) biological process, while the *X* axis represents the number of DEGs in each enriched GO.

Gene ontology (GO) functional enrichment analysis of significantly up-regulated DEGs (Figure 3A) showed that not only genes involved in cellular process like protein synthesis, processing and degradation, but also metabolic activities of the cell, including subcategories of purine nucleotide metabolism, carbohydrate biosynthesis, and energy metabolism presented significant enrichment. Genes encoding various carbohydrate-active enzymes (CAZymes) showed increased levels of transcription, and were included in the hydrolase activity sub-term of catalytic activity term in the GO result, which will be further discussed in the following section. Energy metabolism is important for protein synthesis, and we assume that up-regulation of the related genes may benefit cellulase production in LZ117.

On the other hand, the significantly down-regulated DEGs were mainly enriched in the functional categories of membrane, such as transmembrane transport of anions, carboxylic acids, amino acids and organic acids, and the functional genes involved in some sugars (such as chitin and glucosamine) metabolism and genes belonging to catalytic activity group were also significantly enriched (Figure 3B). We deduce that down-regulation of some key metabolism may be helpful to direct more precursors and energy for enzyme biosynthesis.

**FIGURE 4:**
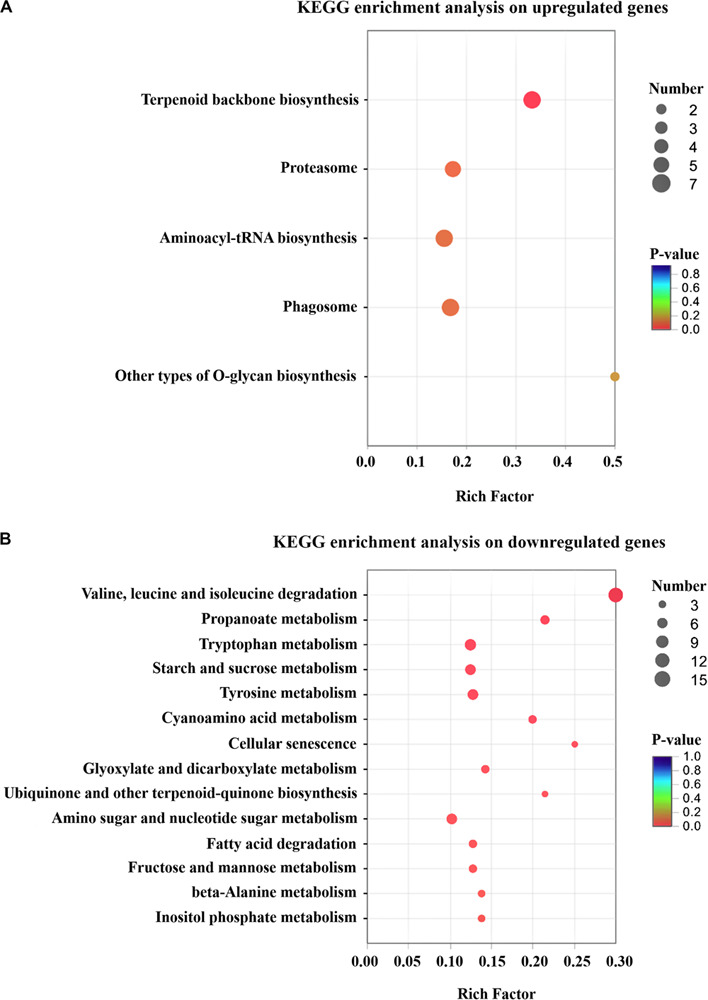
KEGG pathway enrichment analysis of the changed genes. Results of the up-regulated **(A)** and down-regulated **(B)** genes in *T. harzianum* LZ117 relative to *T. harzianum* K223452 were presented. The degree of KEGG enrichment is assessed by the *P*-value and rich Factor. The *X* axis represents Rich factor (the ratio of the number of DEGs (sample numbers) enriched in the pathway to the number of all the annotated genes/transcripts mapped to the corresponding KEGG pathway (Background number). The size of the dot indicates the number of genes in this pathway, and the color of the dot corresponds to a range of different *P*-values (<0.05). The closer the *P*-value is to zero, the greater the Rich factor is, which means that the more the enrichment is significant.

A total of 199 up-regulated genes were involved in cellular process like protein synthesis, processing and degradation, primary metabolism, small molecule metabolism, such as energy metabolism, cofactors, and vitamin metabolism. Furthermore, *T. harzianum* LZ117 may possess more active anabolism of anabolic secondary metabolites considering the DEGs enrichment in the isoprenoid, terpenoid and polyketides biosynthetic metabolic pathways (Figure 4A). Whereas, down-regulated genes were significantly enriched in valine, leucine, and isoleucine degradation, propanoate metabolism, tryptophan metabolism, starch and sucrose metabolism, as well as tyrosine metabolism (Figure 4B). Further exploration of the relationship between these transcriptional changes of pathway genes with cellulase biosynthesis in LZ117 will provide more clues in regulation of cellulase production in this strain.

#### Transcription Analysis of Glycosyl Hydrolase Genes

**FIGURE 5:**
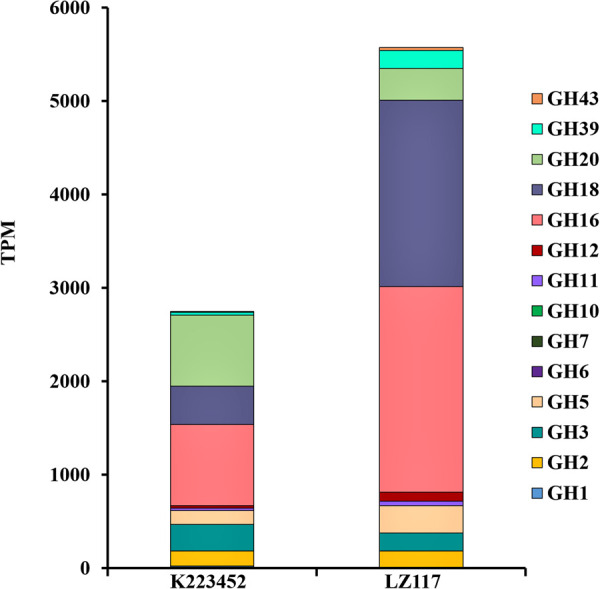
Expression profiles of genes encoding major carbohydrate-active enzymes (CAZymes). The data were extracted from the RNA-seq analysis, which provided the expression values as TPM (Transcripts Per Million reads) mapped for each glycolic hydrolase (GH) family. CAZymes classification was performed according to the annotation of CAzymes genes in *T. harzianum* CBS 226.95 (https://mycocosm.jgi.doe.gov/mycocosm/annotations/browser/cazy/summary;_Jo0wZ?p~=~Triha1).

In view of the maximum productivity of cellulase achieved by *T. harzianum* LZ117 strain at 48 h, transcriptional pattern of the glycoside hydrolase genes in *T. harzianum* LZ117 was illustrated, in which the lignocellulosic degrading-enzymes were focused (Supplementary File S2 and Table 2). As a whole, the transcription levels of 66 CAZyme genes were significantly changed, of which 19 genes were upregulated and 47 genes were downregulated. The expression of glycoside hydrolase genes from GH5, GH6, GH7, GH11, GH12, GH16, and GH18 families were up-regulated in *T. harzianum* LZ117, and the major cellulase genes were distributed into GH5, GH6, and GH7 families. The total transcription quantity of glycoside hydrolase genes belonging to GH3 and GH20 families decreased (Figure 5).

**TABLE 2:** The main DEGs related to (hemi-) cellulases in *T. harzianum* LZ117*.

**Gene ID^a^**	**Gene name**	**Description**	**Log_2_FC^b^**
486211	Mannosidase	GH76 family endo-1,6-alpha-mannosidase	2.91
441083	*egl2*	GH5 family endoglucanase 2	2.90
488374	*cel61b*	GH61 family AA9 protein	2.64
7497	*cbh1*	GH7 family exoglucanase 1	2.54
512303	*egl1*	GH7 family endoglucanase 1	1.33
6750	*cel5b*	GH5 family endo-1,4-glucanase	1.13
513492	*abf*	GH54 family alpha-L-arabinofuranosidase B	1.11
71613	*bgl1*	GH3 family beta-glucosidase (*cel3a*)	1.00
488862	Mannosidase	GH2 family predicted beta-mannosidase	−7.73
72100	Mannosidase	GH76 family endo-1,6-alpha-mannosidase	−5.51
107669	*bgl2*	GH1 family beta-glucosidase (*cel1a*)	−4.74
503269	*abf*	GH54 family alpha-L-arabinofuranosidase B	−4.19
77214	*cel3g*	GH3 family beta-glucosidase (*bgl3i*)	−3.81

*^a^Gene ID was assigned based on the *T. harzianum* CBS 226.95 genome database (https://www.ncbi.nlm.nih.gov/genome/2441?genome_assembly_id~=~370086). **^*b*^**FC: Ratio of the transcription of genes in *T. harzianum* LZ117 over that in *T. harzianum* K223452.

The cellulase cocktail produced by *T. reesei* mainly comprise two cellobiohydrolases (CBHI/Cel7A and CBHII/Cel6A), two endoglucanases (EGI/Cel7B and EGII/Cel5A), and β-glucosidase I (BGLI/Cel3A) that can hydrolyze lignocellulosic complexes synergistically, combined with related hemi-cellulases and auxiliary proteins (Bischof et al., 2016; Druzhinina and Kubicek, 2017). Especially, the major component, CBH1 from *T. reesei* constitutes nearly half of the extracellular protein (Druzhinina and Kubicek, 2017). The transcriptional levels of the major CBH gene *cbh1* and EGs genes *egl1*, *egl2* of *T. harzianum* LZ117 were significantly up-regulated at 24 h comparing with that of K223452 strain, which were essential for robust cellulase production from *T. harzianum* LZ117. Furthermore, the transcription level of *cel5b* gene encoding a GPI anchoring domain-containing membrane-bound protein was also up-regulated in *T. harzianum* LZ117, which is constitutively expressed when cultured on sophorose or lactose, and induced by a small amount under cellulose culture condition in *T. reesei* (Foreman et al., 2003). The transcript of β-glucosidase gene *bgl1* (*cel3a*) in *T. harzianum* LZ117 was increased by around onefold compared with that from *T. harzianum* K223452, which may cause the increased BGL activity of *T. harzianum* LZ117 compared to that of *T. harzianum* K223452. CEL1A has been reported to catalyze transglycosylation of cellobiose to sophorose (sophorose, considered to be the natural strongest inducer of cellulase), which in turn induces *T. reesei* cellulase synthesis (Shida et al., 2015). Surprisingly, the β-glucosidase gene *bgl2* (*cel1a*) of *T. harzianum* LZ117 was down-regulated, whether CEL1A has the similar transglycosylated function as in *T. reesei* for cellulase induction in *T. harzianum* remains to be verified. It is worth noting that the transcription of *cel3g* (*Th_77214*) in *T. harzianum* LZ117 was significantly down-regulated for 14 folds, which may accelerate the initial cellulase induction of *T. harzianum* LZ117, because the present of β-glucosidase gene *cel3g* could affect extracellular lactose absorption and hydrolyze the intracellular sophorose, whose knockout promotes cellulase formation in *T. reesei* (Zou et al., 2018). Increased transcription level of the *cel61b* (*Th_488374*) gene encoding an Auxiliary Activity Family 9 (AA9) protein in *T. harzianum* LZ117 may also help to improve its cellulose hydrolysis efficiency. Even though the transcription level of arabinofuranosidase gene *Th_503269* and two mannosidase genes decreased obviously, no remarkable changes of transcripts of core hemicellulase genes related to xylan degradation in *T. harzianum* LZ117 were observed, such as *xyn1* (*Th_525076*), *xyn2* (*Th_115099*), *xyn3* (*Th_155799*), *axe1* (*Th_94998*), *bxl1* (*Th_502198*), and *cel74a* (*Th_88968*), which resulted in comparable xylanase activities between *T. harzianum* LZ117 and K223452. The downregulated transcripts of gene *Th_133914* encoding extracellular protease (homologous to *tre120998* in *T. reesei*) may contribute to the stability of secreted cellulases from *T. harzianum* LZ117 compared to that in *H. harianum* K223452 (Qian et al., 2019).

#### Changed Genes That Encode Regulators and Major Transporters

The expression of lignocellulose degrading enzymes in filamentous fungi depends on a regulatory network of multiple positive and negative transcription factors (TFs) (Alazi and Ram, 2018). A total of 660 genes in the *T. harzianum* genome were predicted to function as transcriptional regulators, among which the transcripts of 57 genes changed significantly at 24 h, and five of these genes were up-regulated, whereas 52 genes were down-regulated (|Log_2_FC| > 1, *p*-adjust < 0.05) (Supplementary File S3). Besides the regulators Xyr1 (Th_1126) and Cre1 (Th_502975) reported to regulate cellulase production by *T. harzianum* (da Silva et al., 2017), the effects of other differentially expressed transcription factors potentially regulating cellulase induction in *T. harzianum* will be discussed below according to their homologues in *T. reesei*.

In the filamentous fungus *T. reesei*, expression of cellulase is tightly regulated by a series of TFs such as transcriptional activators: Xyr1, Ace3, Vib1 and repressors, including Cre1, Ace1, Rce1, and Ctf1 (Zhang et al., 2018; Liu and Qu, 2019; Meng et al., 2020). Among these factors, the homologues of regulator Xyr1 are the most conserved in cellulolytic fungi (Klaubauf et al., 2014). The C2H2-type TF Cre1/CreA functions as the main negative regulator that mediates carbon catabolite repression (CCR) effect (Li et al., 2015; Huberman et al., 2016; Rassinger et al., 2018). Comparative transcriptome analysis showed that the transcription of *xyr1* (*Th_1126*) gene was significantly up-regulated 1.7-fold in *T. harzianum* LZ117, while the transcriptions of the *cre1* (*Th_502975*), *ace1* (*Th_496062*) and *rce1* (*Th_495839*) genes homologous to counterparts in *T. reesei* were significantly decreased to varying degrees, with fold change (Log_2_FC) from −1.43 <Log_2_FC <−1.97 (Supplementary File S3). However, the transcripts of *ace3, ctf1*, and *vib1* (*Th_140772*, *Th_469719* and *Th_482779*, respectively) have not changed significantly in *T. harzianum* LZ117. The regulator Rxe1 positively modulates the activator Xyr1 and cellulase gene expression in *T. reesei* (Wang et al., 2019). However, the transcript of *rxe1* homolog (*Th_61248*) in *T. harzianum* LZ117 was dramatically decreased, thus the regulatory relationship between Rxe1 and Xyr1 in *T. harzianum* remains unclear. It was also revealed that Xyr1 recruits SWI/SNF complex through direct interactions with TrSNF12 to remodel chromatin at cellulase gene promoters, thereby activating cellulase gene expression in *T. reesei* (Cao et al., 2019) but no significant change for the transcription of *Trsnf12* homolog (*Th_101097*) in *T. harzianum* LZ117 was found as compared to the control strain. The Velvet family protein Ve1 (Liu et al., 2015) in *T. reesei* plays an important role in the regulation of cellulase expression, while transcriptional level of its homologous gene *veA* (*Th_95531*) in *T. harzianum* LZ117 has not changed significantly. These results imply the elevated transcript level of *xyr1* and reduced transcription of *cre1* may partly account for the faster cellulase induction and higher cellulase activity of *T. harzianum* LZ117 in the early phase of cellulase production, and there are different recruitment mechanisms of Xyr1 function between *T. harzianum* LZ117 and *T. reesei*.

Changes in the levels of the main transcription factors Xyr1 and Cre1 proteins under different induced carbon sources may affect the transporter family proteins in *T. reesei*, especially major facilitator superfamily (MFS) (dos Santos et al., 2014, 2016). About 77 genes in the *T. harzianum* genome are predicted to have sugar transport function, and there are about 11 predicted sugar transporter genes with significant transcriptional changes in transcriptome data (|Log_2_FC| > 1, *p*-adjust < 0.05) (Supplementary Table S5). Some sugar transporters have the signal transmitting function for extracellular carbon-induced signals. For example, the sugar transporters Cdt1 and Cdt2 (Znameroski et al., 2013) in *Neurospora crassa*, the sugar transporter CltB (dos Reis et al., 2016) in *A. nidulans*, and the sugar transporter Crt1 in *T. reesei* was reported to possess the function of carbon signaling (Zhang et al., 2013). Unexpectedly, the transcript of *crt1* gene (*Th_502689*) decreased remarkably in *T. harzianum* LZ117 over that in *T. harzianum* K223452. The transcript levels of the homologue of *cdt1* and *cdt2* genes, *Th_75576* and *Th_130330* respectively, in *T. harzianum* LZ117 were also similar to that in K223452, so did the transcript of *cltb* homologous gene (*Th_101977*). Cre-1-mediated CCR in *N. crassa* acts through sugar transporter, transcription factor, sugar catabolism, and PCWDE genes to regulate plant cell wall degradation (Wu et al., 2020). In our results, down-regulation of *cre1* did not correlate with enhanced expressions of the master sugar transporters such as *cdt1*, *cdt2*, which demonstrate that regulatory mechanism underlying Cre1-mediated CCR might be varied between different fungal species. Other novel sugar transporters that are crucial for cellulase induction signaling in *T. harzianum* will be further investigated in future studies.

#### DEGs Related to Protein Synthesis, Sorting and Quality Control

The functional classification analysis for DEGs indicated that the transcriptional levels of 78.6% of the DEGs involved in transcription and protein synthesis in *T. harzianum* LZ117 strains were up-regulated as compared to *T. harzianum* K223452 (Table 3). Specifically, RNA polymerase I, II, and III subunit encoding genes (*Th_71797*, *Th_76852*, and *Th_121495*) were up-regulated by 5.2, 3.4, and 7.2 times, respectively, suggesting that rRNA, mRNA and tRNA synthesis activities the LZ117 strain may be enhanced compared with that in K223452 strain. Regarding the DEGs that function in protein translation process, the transcription of eight genes involved in aminoacyl tRNA synthesis in the LZ117 strain was up-regulated by at least 3.4 times, which provided a sufficient amount of related amino acid donors for the enhanced protein synthesis process of *T. harzianum* LZ117. In addition, up-regulation of the mitochondrial ribosomal protein encoding genes *Th_514053* and *Th_504396* indicate that the energy metabolism of *T. harzianum* LZ117 might be more vigorous than that of K223452. Interestingly, the ribosomal protein encoding genes *Th_113828* and *Th_484754*, and two translation initiation factor encoding genes *Th_8152* and *Th_503429* were significantly up-regulated by at least 3.8 times (Table 3), indicating that protein synthesis of the *T. harzianum* LZ117 may be increased compared to that of K223452.

**TABLE 3:** The DEGs related to protein synthesis in strain LZ117*.

**Gene ID^a^**	**Functional annotation (Swiss-Prot Description)^b^**	**Log_2_FC^c^**
	**Transcription**	
121495	DNA-directed RNA polymerase III subunit RPC10	3.03
71797	DNA-directed RNA polymerase I subunit RPA12	2.63
76852	DNA-directed RNA polymerase II subunit RPB9	2.13
	**Translation**	
72064	Phenylalanine-tRNA ligase	8.30
81396	Putative glycine–tRNA ligase	3.94
115899	Aspartate–tRNA ligase, mitochondrial	3.50
113828	40S ribosomal protein S20	3.30
484440	Glutaminyl-tRNA synthase	3.18
514053	54S ribosomal protein L24, mitochondrial	2.87
513968	Ribonucleoprotein-associated protein	2.78
303	Phenylalanine–tRNA ligase alpha subunit	2.77
482810	Putative proline–tRNA ligase	2.59
503429	Eukaryotic translation initiation factor 5A	2.59
500010	Tyrosine–tRNA ligase, cytoplasmic	2.40
504396	54S ribosomal protein subunit img1, mitochondrial	2.29
8152	Eukaryotic translation initiation factor 3 subunit H	2.27
484754	60S acidic ribosomal protein P2	2.26
479650	Glutamate-tRNA ligase, cytoplasmic	2.13
9281	Translation initiation factor eIF-2B subunit gamma	−2.36

*^a^Gene ID was assigned based on the *T. harzianum* CBS 226.95 genome database (https://www.ncbi.nlm.nih.gov/genome/2441?genome_assembly_id=370086). **^*b*^**Annotation of RNA-seq data against Swiss-Prot database. **^*c*^**FC: Ratio of the transcription of genes in strain LZ117 over that in strain K223452.

Regarding the protein folding processing and sorting pathway (Table 4), the transcription of the glucosidase II β subunit-encoding gene (*Th_83575*) in *T. harzianum* LZ117 strain was significantly up-regulated, which may be involved in protein glycosylation modification in the endoplasmic reticulum (Cui et al., 2016). In addition, in *T. harzianum* LZ117, the transcription levels of gene *Th_482508* encoding the molecular chaperone T complex 1β subunit, and gene *Th_92965* encoding the signal recognition particle receptor α subunit were up-regulated by 6.2- and 5.0-fold respectively, which are beneficial for protein folding and exosome-based protein secretion (Wu et al., 2015). Furthermore, significant changes were also observed in genes related to chaperones and ubiquitin-proteasome system for protein quality control (Table 4), the transcription of *hacI* gene (*Th_72005*) in *T. harzianum* LZ117 was 1.7 times that of the corresponding transcript of *T. harzianum* K223452, and the transcription of *Th_510360* encoding heat shock protein Hsp30 was up-regulated by 5.0-fold. Meanwhile, the transcription levels of ubiquitin-activating enzyme E1 gene *Th_6117* responsible for misfolded protein degradation, the ubiquitin homeostasis regulatory protein encoding gene *Th_488956*, and genes encoding multiple proteasome subunits were significantly up-regulated. The changes of endoplasmic reticulum marker enzymes and ubiquitin-proteasome proteins indicated increased cellulase synthesis by *T. harzianum* LZ117 at the early cellulase production stage.

**TABLE 4:** The DEGs related to protein folding, sorting and degradation in strain LZ117*.

**Gene ID^a^**	**Functional annotation (Swiss-Prot Description)^b^**	**Log_2_FC^c^**
83575	Glucosidase II beta subunit	4.33
352623	Proteasome subunit alpha type-6	2.93
482508	Probable T-complex protein 1 subunit beta	2.85
92409	Proteasome subunit beta type-1	2.81
488956	Ubiquitin homeostasis protein	2.66
504589	Proteasome subunit beta type-3	2.63
92965	Signal recognition particle receptor subunit alpha	2.58
510360	Heat shock protein Hsp30	2.58
480080	Proteasome subunit alpha type-7	2.41
505979	Protein transport protein sec13	2.37
511826	Proteasome subunit alpha type-3	2.22
6117	Ubiquitin-activating enzyme E1	2.18
405376	26S proteasome regulatory subunit	2.12
145858	Endoplasmic reticulum chaperone	−6.54

*^a^Gene ID was assigned based on the *T. harzianum* CBS 226.95 genome database (https://www.ncbi.nlm.nih.gov/genome/2441?genome_assembly_id~=~370086). **^*b*^**Annotation of RNA-seq data against Swiss-Prot database. **^*c*^**FC: Ratio of the transcription of genes in strain LZ117 over that in strain K223452.

Previously, transcriptomic profile analysis of *T. harzianum* underlying biomass degradation has been conducted using *T. harzianum* IOC-3844 induced by sugarcane bagasse and cellulose (Horta et al., 2014). In a recent study, transcriptome of *T. harzianum* was compared with other two *Trichoderma* species (Horta et al., 2018). However, the samples in both studies were collected at 96 h. In the current study, a global transcriptional profile involved in the induction and transcriptional regulation of cellulase was focused, which contributes to the catalog of publicly available transcriptome data from *T. harzianum*, and provides useful clues for unraveling the biotechnological potential of this important *Trichoderma* species for lignocellulosic biorefinery. Genes identified in this study can be further analyzed and used to improve cellulase production in other *T. harzianum* strains as well as *T. reesei* strains. Further studies on cellulase production from *T. harzianum* will unveil more important aspects of this species and enrich the knowledge on cellulase production by filamentous fungi.

In this report, comprehensive analysis of global gene transcription in the promising cellulase producing strain LZ117 at early cellulase-producing stage has been performed. However, the possibility that other mechanisms also exist that affect cellulase production in this strain cannot be excluded, including single nucleotide polymorphism in the genome, post-translational control, and so on. The finding in this study is a reminder that we still have much to learn on the potential of biotechnology applications of microbial strains from Tibet in China, and provide a basis for further exploration of other fungal enzyme producers from specific environments.

## Conclusion

In this study, 88 filamentous fungal strains were isolated from 36 samples collected from Tibet. Screening for cellulase-producing fungi indicated *T. harzianum* LZ117 is the most potent strain. Comparative transcriptome analysis between *T. harzianum* LZ117 and the control strain revealed significant modulation and quality control of protein synthesis, processing and degradation in *T. harzianum* LZ117 at early cellulase producing stage. Up-regulation of the activator Xyr1 in combination with down-regulation of the repressor Cre1 may lead to early induction of glycoside hydrolases, and global changes in transcriptional regulation, protein production and processing may also contribute to high cellulase production titer in *T. harzianum* LZ117.

## Data Availability Statement

The datasets presented in this study can be found in online repositories. The names of the repository/repositories and accession number(s) can be found in the article/Supplementary Material.

## Author Contributions

J-XL and FZ performed the experiments and analyzed the data. D-DJ, JL, F-LW, and ZZ provided the samples from Tibet and participated in discussion of strain identification. J-XL drafted the manuscript. X-QZ designed the experiments and critically revised the manuscript. WW participated in discussion of the data and revised the manuscript. All the authors read and approved the final version of the manuscript.

## Conflict of Interest

D-DJ, JL, F-LW, and ZZ are employed by JALA Group Co.

The remaining authors declare that the research was conducted in the absence of any commercial or financial relationships that could be construed as a potential conflict of interest.

## Footnotes

1 https://blast.ncbi.nlm.nih.gov/Blast.cgi

2 https://genome.jgi.doe.gov/mycocosm/home

3 http://www.ebi.ac.uk/Tools/msa/clustalo/

4 https://www.ncbi.nlm.nih.gov/genome/2441?genome_assembly_id=370086

5 https://mycocosm.jgi.doe.gov/mycocosm/annotations/browser/cazy/summary;_Jo0wZ?p=Triha1

6 https://mycocosm.jgi.doe.gov/pages/search-for-genes.jsf?organism=Trire2

7 www.majorbio.com

8 www.ncbi.nlm.nih.gov/sra/
