# Supplementary material for: Diversity of Cellulase-Producing Filamentous Fungi From Tibet and Transcriptomic Analysis of a Superior Cellulase Producer Trichoderma harzianum LZ117
Source: Front Microbiol. 2020 Jul 14;11:1617. doi: 10.3389/fmicb.2020.01617 (PMC7372938; doi:10.3389/fmicb.2020.01617)
Supplement: Supplementary file 1 [file Table_1.DOCX]

**Supplementary materials for the manuscript by Jiaxiang Li et al.**

**Supplementary Tables**

**Table S1** Filamentous fungal species identified in this study

| Culture code | Origin | | Organism identification | NCBI Accession No. (ITS) |  |  |  |  |  |
| --- | --- | --- | --- | --- | --- | --- | --- | --- | --- |
|  | Isolation source | Localization |  |  |  |  |  |  |  |
| SC2-75 | *Cotoneaster microphyllus Lindl.* (**plant**) | (94°30'17''E, 29°33'59''N) **Altitude: 3080m** | *Fusarium* sp. | MK804369 |  |  |  |  |  |
| SC2-81 |  |  | *Fusarium* sp. | MK804375 |  |  |  |  |  |
| SC2-112 |  |  | *Curvularia* sp. | MK804384 |  |  |  |  |  |
| SC3-48 | *Sambucus adnata Wall.*  (**plant**) | (94°30'26''E, 29°33'33''N) **Altitude: 3670m** | *Aspergillus* sp. | MK804350 |  |  |  |  |  |
| SC3-49 |  |  | *Aspergillus* sp. | MK804351 |  |  |  |  |  |
| SC3-116 |  |  | *Peyronellaea* sp. | MK804387 |  |  |  |  |  |
| SC3-DY9 |  |  | *Aspergillus* sp. | MK804403 |  |  |  |  |  |
| SC4-73 | *Polygonum polystachyum Wall.* (**plant**) | (94°30'26''E, 29°33'33''N) **Altitude: 3670m** | *Fusarium* sp. | MK804368 |  |  |  |  |  |
| SC4-77 |  |  | *Fusarium* sp. | MK804371 |  |  |  |  |  |
| SC4-78 |  |  | *Fusarium* sp. | MK804372 |  |  |  |  |  |
| SC10-67 | *Gentiana obconica* (**plant**) | (94°38'35''E, 29°37'04''N) **Altitude: 4520m** | *Mucor* sp. | MK804366 |  |  |  |  |  |
| SC11-5 | Unknown mushroom (**fungi**) | (94°38'35''E, 29°37'04''N) **Altitude: 4520m** | *Mucor* sp. | MK804325 |  |  |  |  |  |
| SC11-65 |  |  | *Trichoderma* sp. | MK804364 |  |  |  |  |  |
| SC12-35 | Lichen on stone surface  (**plant**) | (94°38'35''E, 29°37'04''N) **Altitude: 4520m** | *Fusarium* sp. | MK804348 |  |  |  |  |  |
| SC12-72 |  |  | *Fusarium* sp. | MK804367 |  |  |  |  |  |
| SC12-118 |  |  | *Paecilomyces* sp. | MK804389 |  |  |  |  |  |
| SC12-DY54 |  |  | *Fusarium* sp. | MK804402 |  |  |  |  |  |
| SC13-3 | Lichen parasitic on the azalea (**plant**) | (94°38'35''E, 29°37'04''N) **Altitude: 4520m** | *Mucor* sp. | MK804323 |  |  |  |  |  |
| SC13-55 |  |  | *Psathyrella* sp. | MK804354 |  |  |  |  |  |
| SC13-114 |  |  | *Trichoderma* sp. | MK804386 |  |  |  |  |  |
| SC15-L7 | White linear lichen on the earth's surface (**plant**) | (94°38'35''E, 29°37'04''N) **Altitude: 4520m** | *Penicillium* sp. | MK804408 |  |  |  |  |  |
| SC15-25 |  |  | *Mucor* sp. | MK804342 |  |  |  |  |  |
| SC15-95 |  |  | *Mucor* sp. | MK804379 |  |  |  |  |  |
| SC15-DY1 |  |  | *Penicillium* sp. | MK804392 |  |  |  |  |  |
| SC15-DY4 |  |  | *Penicillium* sp. | MK804400 |  |  |  |  |  |
| SC15-DY53 |  |  | *Trichoderma* sp. | MK804401 |  |  |  |  |  |
| CGMCC-LZ117 | Moss on stone surface (**plant**) | (94°38'35''E, 29°37'04''N) **Altitude: 4520m** | *Trichoderma* harzianum | MK804404 |  |  |  |  |  |
| SC17-98 |  |  | *Cladosporium* sp. | MK804380 |  |  |  |  |  |
| SC17-99 |  |  | *Alternaria* sp. | MK804381 |  |  |  |  |  |
| **Table S1 (*continued*)** | |  |  |  |  |  |  |  |  |
| Culture code | Origin |  | Organism identification | NCBI Accession No. (ITS) |  |  |  |  |  |
|  | Isolation source | Localization |  |  |  |  |  |  |  |
| SC18-123 | *Saussurea obvallata* (**plant**) | (94°38'35''E, 29°37'04''N) **Altitude: 4520m** | *Aspergillus* sp. | MK804390 |  |  |  |  |  |
| SC19-15 | Meltwater from the high mountains (**aquatic sample**) | (94°33'59''E, 29°34'07''N) **Altitude: 3530m** | *Mucor* sp. | MK804334 |  |  |  |  |  |
| SC21-31 | *Sambucus adnata Wall* (**plant**) | (94°33'59''E, 29°34'07''N) **Altitude: 3530m** | *Fusarium* sp. | MK804346 |  |  |  |  |  |
| SC23-58 | *Rosa platyacantha Schrenk* (**plant**) | (94°33'59''E, 29°34'07''N) **Altitude: 3530m** | *Arthrinium* sp. | MK804357 |  |  |  |  |  |
| SC23-53 |  |  | *Fusarium* sp. | MK804353 |  |  |  |  |  |
| SC23-61 |  |  | *Phaeophlebiopsis* sp. | MK804360 |  |  |  |  |  |
| SC26-62 | *Rosa macrophylla Lindl. var. glandulifera* (**plant**) | (94°33'59''E, 29°34'07''N) **Altitude: 3530m** | *Thyronectria* sp. | MK804361 |  |  |  |  |  |
| SC27-63 | *Epilobium angustifolium L.* (**plant**) | (94°42'32''E, 29°37'41''N) **Altitude: 4180m** | *Psathyrella* sp. | MK804362 |  |  |  |  |  |
| SC28-6 | *Fragaria nubicola (Hook. f.) Lindl. ex Lacaita* (**plant**) | (94°42'32''E, 29°37'41''N) **Altitude: 4180m** | *Mucor* sp. | MK804326 |  |  |  |  |  |
| SC30-80 | Unknow rose (**plant**) | (94°42'32''E, 29°37'41''N) **Altitude: 4180m** | *Fusarium* sp. | MK804374 |  |  |  |  |  |
| SC32-4 | Parasite on bark (**plant**) | (94°41'48''E, 29°38'31''N) **Altitude: 4030m** | *Mucor* sp. | MK804324 |  |  |  |  |  |
| SC32-56 |  |  | *Mucor* sp. | MK804355 |  |  |  |  |  |
| SC35-13 | Moss on stone surface (**plant**) | (94°41'48''E, 29°38'31''N) **Altitude: 4030m** | *Mortierella* sp. | MK804332 |  |  |  |  |  |
| SC35-66 |  |  | *Mucor* sp. | MK804365 |  |  |  |  |  |
| SC35-83 |  |  | *Mucor* sp. | MK804376 |  |  |  |  |  |
| SC37-29 | Unknown mushroom (**fungi**) | (94°41'48''E, 29°38'31''N) **Altitude: 4030m** | *Mucor* sp. | MK804345 |  |  |  |  |  |
| SC43-12 | *Gentiana obconica* (**plant**) | (94°47'37''E, 29°40'01''N) **Altitude: 4480m** | *Fusarium* sp. | MK804331 |  |  |  |  |  |
| SC49-117 | Leaves from *Rheum nobile* (**plant**) | (94°47'16''E, 29°40'10''N) **Altitude: 4410m** | *Cladosporium* sp. | MK804388 |  |  |  |  |  |
| SC55-108 | *Gentiana veitchiorum Hemsl.* (**plant**) | (94°44'37''E, 29°39'47''N) **Altitude: 4018m** | *Alternaria* sp. | MK804382 |  |  |  |  |  |
| SC56-76 | *Gentiana veitchiorum Hemsl.* (**plant**) | (94°43'40''E, 29°36'44''N) **Altitude: 3610m** | *Fusarium* sp. | MK804370 |  |  |  |  |  |
| SC56-52 |  |  | *Robillarda* sp. | MK804352 |  |  |  |  |  |
| SC56-109 |  |  | *Phoma* sp. | MK804383 |  |  |  |  |  |
| SC56-113 |  |  | *Trichoderma* sp. | MK804385 |  |  |  |  |  |
| SB17-DY36 | Tibet highland **barley wine starter** | Tibet starter, Tibetan plateau. | *Coniochaeta* sp. | MK804394 |  |  |  |  |  |
| SB17-DY37 |  |  | *Phomatospora* sp. | MK804395 |  |  |  |  |  |
| SB17-DY40 |  |  | *Mucor* sp. | MK804396 |  |  |  |  |  |
| **Table S1 (*continued*)** | | | | |  |  |  |  |  |
| Culture code | Origin | | Organism identification | NCBI Accession No. (ITS) |  |  |  |  |  |
|  | Isolation source | Localization |  |  |  |  |  |  |  |
| SB17-DY42 | Tibet highland **barley wine starter** | Tibet starter, Tibetan plateau. | *Cytospora* sp. | MK804397 |  |  |  |  |  |
| SB17-DY46 |  |  | *Mucor* sp. | MK804398 |  |  |  |  |  |
| SB17-DY49 |  |  | *Talaromyces* sp. | MK804399 |  |  |  |  |  |
| A01-L8 | **Soil**, Mountain Shegyla | (94°38'26''E, 29°37'01''N) **Altitude: 4500m** | *Talaromyces* sp. | MK804409 |  |  |  |  |  |
| A01-22 |  |  | *Mortierella* sp. | MK804339 |  |  |  |  |  |
| A02-DY17 | **Soil**, Mountain Shegyla | (94°38'26''E, 29°37'01''N) **Altitude: 4530m** | *Mucor* sp. | MK804391 |  |  |  |  |  |
| A03-16 | **Soil**, Mountain Shegyla | (94°38'26''E, 29°37'01''N) **Altitude: 4540m** | *Mucor* sp. | MK804335 |  |  |  |  |  |
| A03-17 |  |  | *Mortierella* sp. | MK804336 |  |  |  |  |  |
| A03-18 |  |  | *Fusarium* sp. | MK804337 |  |  |  |  |  |
| A03-79 |  |  | *Fusarium* sp. | MK804373 |  |  |  |  |  |
| A04-21 | **Soil**, Mountain Shegyla | (94°38'26''E, 29°37'01''N) **Altitude: 4560m** | *Mortierella* sp. | MK804338 |  |  |  |  |  |
| A04-26 |  |  | *Mucor* sp. | MK804343 |  |  |  |  |  |
| A04-92 |  |  | *Fusarium* sp. | MK804378 |  |  |  |  |  |
| A04-DY23 |  |  | *Penicillium* sp. | MK804393 |  |  |  |  |  |
| A05-23 | **Soil**, Mountain Shegyla | (94°38'32''E, 29°37'07''N) **Altitude: 4560m** | *Mucor* sp. | MK804340 |  |  |  |  |  |
| A05-34 |  |  | *Tolypocladium* sp. | MK804347 |  |  |  |  |  |
| A05-42 |  |  | *Tolypocladium* sp. | MK804349 |  |  |  |  |  |
| A05-57 |  |  | *Mucor* sp. | MK804356 |  |  |  |  |  |
| A05-59 |  |  | *Mucor* sp. | MK804358 |  |  |  |  |  |
| A06-28 | **Soil**, Mountain Shegyla | (94°39'05''E, 29°26'50''N) **Altitude: 4590m** | *Mucor* sp. | MK804344 |  |  |  |  |  |
| A06-64 |  |  | *Penicillium* sp. | MK804363 |  |  |  |  |  |
| B01-7 | **Soil**, Mountain Shegyla | (94°42'32''E, 29°37'41''N) **Altitude: 4190m** | *Mucor* sp. | MK804327 |  |  |  |  |  |
| B01-8 |  |  | *Mortierella* sp. | MK804328 |  |  |  |  |  |
| B01-14 |  | (94°42'32''E, 29°37'41''N) **Altitude: 4190m** | *Mucor* sp. | MK804333 |  |  |  |  |  |
| B02-1 | **Soil**, Mountain Shegyla | (94°41'49''E, 29°38'31''N) **Altitude: 4030m** | *Mortierella* sp. | MK804322 |  |  |  |  |  |
| B02-9 |  |  | *Mortierella* sp. | MK804329 |  |  |  |  |  |
| C01-L2 | **Soil**, Dongbacai Village | (94°47'36''E, 29°40'00''N) **Altitude: 4510m** | *Trichoderma* sp. | MK804405 |  |  |  |  |  |
| C03-11 | **Soil**, Lulang Flower Sea | (94°44'40''E, 29°36'43''N) **Altitude: 3630m** | *Hypocrea* sp. | MK804330 |  |  |  |  |  |
| C03-24 |  |  | *Hypocrea* sp. | MK804341 |  |  |  |  |  |
| C03-60 |  |  | *Hypocrea* sp. | MK804359 |  |  |  |  |  |
| C03-87 |  |  | *Penicillium* sp. | MK804377 |  |  |  |  |  |
| C03-L3 |  |  | *Trichoderma* sp. | MK804406 |  |  |  |  |  |
| C03-L4 |  |  | *Trichoderma* sp. | MK804407 |  |  |  |  |  |

**Table S2** Components in Mediums used in this study for fungal isolation.

| **Mediums** | **Components (w/v)** |
| --- | --- |
| PDA | 20% Potato, 2% Glucose, 1.5% Agar |
| Malt extract agar (MEA) | 3% Malt extract, 1.5% Agar |
| Martin agar | 0.5% Peptone, 1% Glucose, 0.1% KH_2_PO_4_, 0.05% MgSO_4_∙7H_2_O,  1.5% Agar |
| Honey agar | 1% Yeast extract, 2% Honey, 2% Peptone, 1.5% Agar |
| CZA | 3% Sucrose, 0.3% NaNO_3_, 0.1% K_2_HPO_4_, 0.05% KCl,  0.05% MgSO_4_∙7H_2_O, 0.001% FeSO_4_, 1.5% Agar |
| YPD | 1% Yeast extract, 2% Glucose, 2% Peptone, 1.5% Agar |
| G25N | 0.1% KH_2_PO_4_, 0.05% KCl, 0.05% MgSO_4_∙7H_2_O, 0.001% FeSO_4_∙7H_2_O, 0.001% ZnSO_4_, 0.0005% CuSO_4_∙7H_2_O, 0.37% Yeast extract,  25% Glycerol, 1.5% Agar |
| Sabouraud medium | 1% Peptone, 2% Agar, 4% Maltose |
| Rice water media | Rice porridge (10%) filtrate supplemented with 1.5% Agar |
| Mandels-Andreotti (MA) | 0.14% (NH_4_)_2_SO_4_, 0.2% KH_2_PO_4_, 0.03% MgSO_4_∙7H_2_O,  0.04% CaCl_2_∙2H_2_O, 0.00005% FeSO_4_∙7H_2_O, 0.00017% MnSO_4_∙H_2_O, 0.00014% ZnSO_4_∙7H_2_O, 0.00002% CoCl_2_, 0.1% Peptone,  0.2 M Na_2_HPO_4_-Citric acid buffer (50% (v/v), pH 5.0),  2% Carbon source |

**Table S3** Primers used in this study

| **Primers** | **Sequences (5’-3’)** | **Relevant genes** |
| --- | --- | --- |
| **Cloning of CDS sequences of focused genes** | |  |
| Thxyr1-F | TCTCAATCCCATCTCCACTCAATAAA | *xyr1* |
| Thxyr1-R | AACGTATGGAGACATTGGGTGCA |  |
| Thcre1-F | AGGACAGGACAGCGACGCCGAGCAA | *cre1* |
| Thcre1-R | CCACCTTGGGAACCTGAGGAACA |  |
| Thcbh1-F | CCACCTCTGACATGGCAGAAATGC | *cbh1* |
| Thcbh1-R | AGCTTCGACTTGGGCGGGAACAC |  |
| ThEGII-F | GCTACTTTACTGGCTGGCGGCTCTA | *egl2* |
| ThEGII-R | CGGAGCCAAGTGGCAACAGGTGA |  |
| ThEGI-F | GCACTCCCGAGGTCCATCCCAAGT | *egl1* |
| ThEGI-R | GCTTGAAGTGGAAGTTCTCAGGGTC |  |
| Thbgl1-F2 | AGACKCCTCAGGGAGGTCGNAAC | *bgl1* |
| Thbgl1-R2 | TNTGCGANCTCGTATCCCAGTAG |  |
| Thβ-tub-F | CCGGCCAGTGCGGTAACCAAATCG | *β-tubulin* |
| Thβ-tub-R | TCCTGGTATTGCTGGTACTCAGACAC |  |
| **RT-qPCR** | |  |
| qXyr1-F | ACAGGGACTACTGGGCCAATC | *xyr1* |
| qXyr1-R | AATGGGCACAAGGATGTAAACC |  |
| qCre1-F | TCACACTCCCATCGCAACTC | *cre1* |
| qCre1-R | CGTGTTGTGCTGCAGAGACA |  |
| qCBH1-F | CCTGATGGCTGCGACTGGAAC | *cbh1* |
| qCBH1-R | AACGGTCAACTTCTTGGTGGTGTC |  |
| qEGI-F | AAGCGACTTGTGGTGCCAACTG | *egl1* |
| qEGI-R | CTCCTGACCGTTCAACTGAAGCAG |  |
| qEGII-F | CCACCTAGCTCTGGCCTCACC | *egl2* |
| qEGII-R | CGAAGTGACGCAGGTTCCATCAG |  |
| qBGL1-F | GTCGCCTCTGTCATGTGCTCTTAC | *bgl1* |
| qBGL1-R | GCCTGCACCGTCGAGTGTTG |  |
| qTubulin-F | TTCATCTTCGGCCAATCCTCTGC | *β-tubulin* |
| qTubulin-R | CTCACGGCGGATCACGTCAAG |  |

**Table S4 Major database used for gene function annotation**

| **Databases** | **Version** | **Sources** |
| --- | --- | --- |
| NR | Ver. 2019.6 | ftp://ftp.ncbi.nlm.nih.gov/blast/db/ |
| GO | - | http://www.geneontology.org/ |
| KEGG | Ver. 2017.8 | http://www.genome.jp/kegg/ |
| eggNOG | Ver. 5.0 | http://eggnogdb.embl.de/#/app/home |
| Pfam | Ver. v32.0 | http://pfam.xfam.org/ |
| Swiss-prot | Ver. 2019.7 | ftp://ftp.uniprot.org/pub/databases/uniprot/ |

**Table S5** The transcriptional changes of predicted sugar transporter genes in *T. harzianum* LZ117^*^

| **Gene ID^a^** | **Functional description** | **Log_2_FC^b^** |
| --- | --- | --- |
| 480915 | Predicted transporter (major facilitator superfamily) | 3.29 |
| 127788 | Predicted transporter (major facilitator superfamily) | -8.97 |
| 99293 | Predicted transporter (major facilitator superfamily) | -6.06 |
| 93498 | Predicted transporter (major facilitator superfamily) | -5.25 |
| 82579 | Predicted transporter (major facilitator superfamily) | -5.15 |
| 78790 | Predicted transporter (major facilitator superfamily) | -4.91 |
| 90805 | Predicted transporter (major facilitator superfamily) | -4.05 |
| 86552 | Predicted transporter (major facilitator superfamily) | -3.67 |
| 119756 | Predicted transporter (major facilitator superfamily) | -3.12 |
| 89227 | Predicted transporter (major facilitator superfamily) | -2.91 |
| 502689 | Predicted transporter (major facilitator superfamily) | -2.51 |

^*^**^a^**Gene ID was assigned based on the *T. harzianum* CBS 226.95 genome database (https://www.ncbi.nlm.nih.gov/genome/2441?genome_assembly_id=370086)

**^b^**FC: Ratio of the transcription of genes in *T. harzianum* LZ117 over that in *T. harzianum* K223452*.*

**Supplementary figures**

**
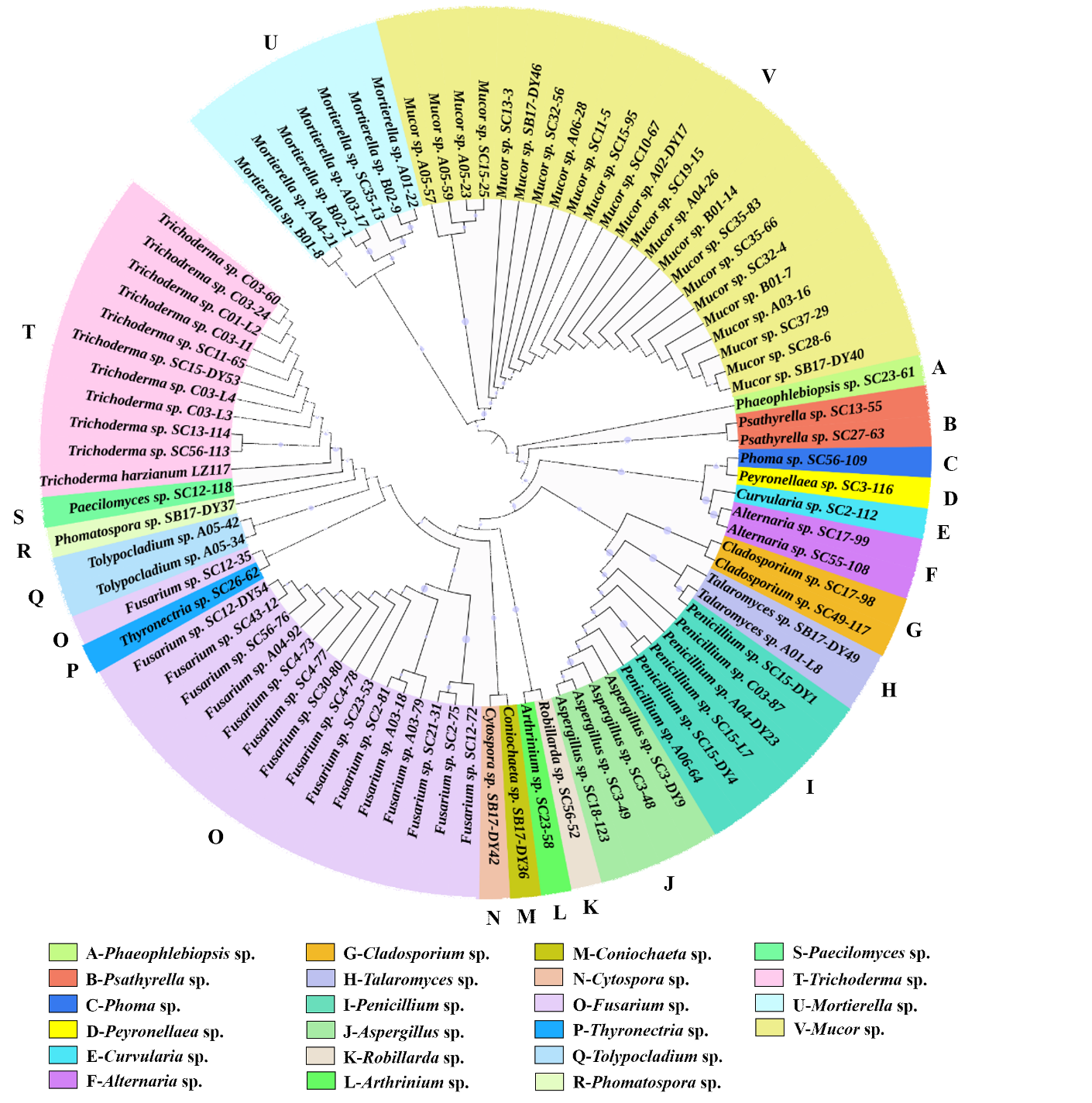
**

**Figure S1** Neighbor-joining phylogenetic tree of the isolated fungal strains.

The phylogenetic tree was constructed based on ITS sequences of the strains.


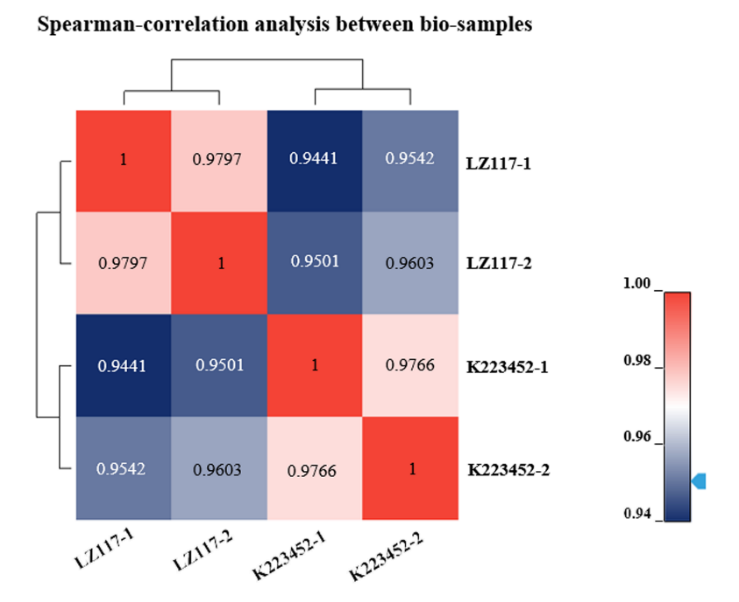


Ad


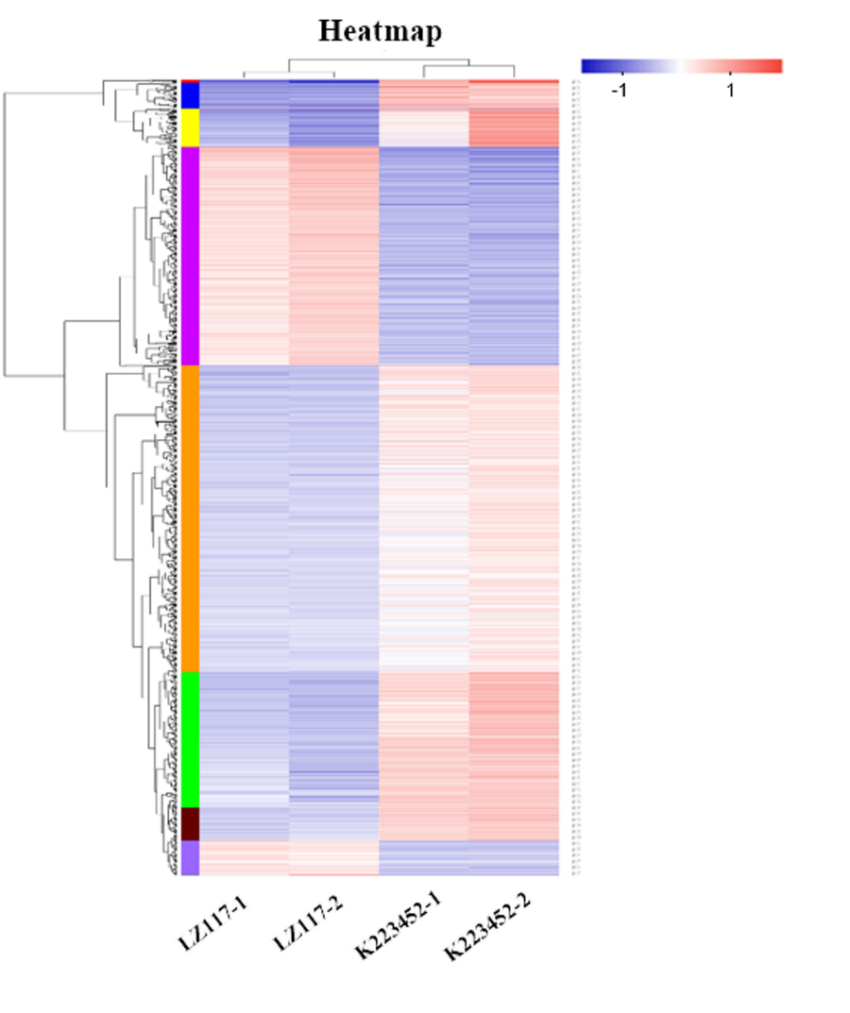


Bd

**Figure S2** Spearmen-correlation analysis of the transcriptome data between the sample replicates (A) and Clustering heat map of gene expression pattern of the transcriptome data (B)^*^

^*^LZ117-1 and LZ117-2 indicate two biological replicates of strain LZ117, K223452-1 and K223452-2 are two replicates of strain K223452.

**List of Additional files (Please find in the folder as Excel files)**

**Additional file 1.** DEGs between *T. harzianum* LZ117 and *T. harzianum* K223452

**Additional file 2.** Transcriptional pattern changes of CAZymes in *T. harzianum* LZ117 over that in *T. harzianum* K223452

**Additional file 3.** DEGs functioning as regulators between *T. harzianum* LZ117 and *T. harzianum* K223452
